# Multilevel selection in multitype populations

**DOI:** 10.1093/pnasnexus/pgag180

**Published:** 2026-05-20

**Authors:** Amanda de Azevedo-Lopes, Arne Traulsen

**Affiliations:** Department of Theoretical Biology, Max Planck Institute for Evolutionary Biology, August-Thienemann Str. 2, 24306 Plön, Schleswig-Holstein, Germany; Department of Theoretical Biology, Max Planck Institute for Evolutionary Biology, August-Thienemann Str. 2, 24306 Plön, Schleswig-Holstein, Germany

**Keywords:** biological interactions, microbiome, evolutionary game theory

## Abstract

Multilevel selection has important implications for understanding the origin, ecology, and evolution of host-associated microbiomes. Selection on the host-level can have a substantial impact on the evolution of microbial lineages, favoring microbes that are beneficial to the host. However, previous research has focused on the evolution of interactions among only two types. We alter this perspective by examining the role of multilevel selection in shaping the interaction dynamics of a population with many microbial types—a case of particular relevance for microbiomes. We ask how multilevel selection influences the selection of interactions among various microbial types, whether it promotes microbial diversity within the population, and whether it increases the likelihood of microbial lineages evolving beneficial interactions with their host and other microbes. To address these questions, we simulate a multitype population structured into groups, where individuals interact within groups through an evolutionary game that determines their fitness. We classify pairwise interactions by their dynamical outcomes: dominance, coexistence, or bistability. We find that multilevel selection reshapes interactions dynamics in complex ways, depending on the details of the population structure. We show the impact of the interaction patterns emerging in such a system.

Significance statement Multilevel selection has attracted considerable attention in the context of host-associated microbiomes, as microbiome composition has been shown to influence host fitness. Multilevel selection theory is only concerned with the evolution of cooperation between two types of individuals, arguing about the relative strengths of selection on the different levels. However, often there are many different types on the lower level that cannot be classified only as cooperators and non-cooperators. In particular, in the context of microbiome dynamics, populations have a very large number of microbial types. Here, we ask how such communities are shaped by selection on the higher level, focusing on the interactions among them.

## Introduction

Microbial communities can be complex, composed of many species interacting in intricate ways (1). Interactions form and shape the structure and dynamics of these communities (2, 3), leading to emergent properties that cannot be described only by the sum of the properties of a collection of species (4, 5). To date, we do not have a clear understanding of the different levels that can affect selection on microbial communities. At the individual level, interactions between microbes vary influenced by biological, chemical, and physical factors (1, 6, 7). At the community level, various functional properties emerge, and selection may favor communities with specific functions. Selection can act in opposite directions at the individual and the community level (5, 8). At an even higher level, microbial communities can form complex associations with hosts, such as microbiomes, which in turn influences host fitness and health (9, 10). The study of microbiomes has grown into a major research field, with its focus ranging from the origin to the functions of these associations, which may impact the host development, lifespan, and reproductive success (9–11). Therefore, selection at a higher level could favor microbes with traits that are costly to themselves if they provide a benefit to their host. Thus, multilevel selection has important implications for understanding the origin and evolution of the association between host-associated microbiomes (12).

Multilevel selection has been widely studied mathematically in the context of evolutionary dynamics (12–22). These studies have investigated the effects of higher-level selection on the evolution of interactions, particularly in the context of evolution of cooperation and the conditions under which cooperation can be sustained. For example, van Vliet and Doebeli (19) demonstrated that coevolution between a host and its microbiome can be maintained when the host’s generation time is short and microbial inheritance (vertical transmission) is stronger than environmental migration (horizontal transmission). While some of these studies acknowledge interactions involving more than two types of individuals, they analyze interactions between two types of individuals (13–15, 17, 19–21). Yet natural microbial communities are far more complex, and typically contain multiple types of individuals with different types of interactions between them.

In this article, we focus on the role of multilevel selection in shaping the interactions in such a population with many microbial types. Ecological interactions are thought to influence the diversity of a community and species coexistence in different ways (23–25). These interactions are influenced by biotic and abiotic conditions, such as mechanisms by which microbes interact, nutrient availability, and temperature (26–28). Often they are context dependent, such as when the interaction between two species change in the presence or absence of other species. For example, under low availability of environmental resources, some individuals may compete with each other, while others may form positive associations. In addition, historical contingency can matter, eg the order in which individuals arrive in the community may also promote or hinder the growth of other individuals (29, 30).

We develop a model to start bridging the gap between abstract multilevel selection models with two types and natural microbial communities with many types. We consider a population of *d* types of individuals structured into *m* groups. Groups can be interpreted as hosts and individuals as microbial types, mimicking a host-associated microbial community. Individuals interact with other group members through an evolutionary game that determines their fitness. Groups can split if they exceed a maximum size *n*, and individuals will be randomly distributed into the two new groups. To keep the number of groups constant, when one group divides, another one is removed. Multilevel selection emerges as a consequence of individual reproduction and constraint from population structure. For two types, it has been shown that cooperation can be favored in this setup (15). Mechanisms that help promote cooperation are widely studied in the context of evolutionary dynamics, and often interspecies cooperation and mutualism are used as synonyms (31, 32). It has recently been argued that these analogies between game theoretical classification of interactions, such as cooperation, and ecological classification of interactions, such as mutualisms, are not always appropriate (33). As even the classification of interspecies cooperation in this context is not straightforward, we focus on the game theoretical classification of interactions, equivalent to their dynamical outcomes (33). We classify pairwise interactions between types based on their dynamical outcomes, rather than focusing on which types of individuals are selected, as an individual that can be in a mutualism with one type could be a predator for another. We analyze the effect of multilevel selection on the evolution of these interactions.

We show that multilevel selection influences the selection of interactions. We explore how they depend on the number of groups, the maximum group size, and the group splitting probability. In particular, we show that the fraction of bistable interactions is increased in group-structured populations, whereas the fraction of coexistence interactions is decreased. In addition, we analyze different measures of diversity and discuss the impact of the group structure in maintaining and promoting diversity in the population.

## Model

We consider a population with *d* types distributed into *m* groups, each with a carrying capacity of *n* individuals. Thus, the maximal population size is N=mn. Individuals interact only within their group through an evolutionary game that determines their fitness. The payoff of a type *j* in group *g* is given by


(1) $$\pi_{j}({\vec{n}}^{g})=\frac{n_{j}^{g}-1}{N^{g}-1}a_{\;jj}+\sum_{\begin{array}{c} k=1,\\ k\neq j \end{array}}^{d}\frac{n_{k}^{g}}{N^{g}-1}a_{\;jk},$$

where each individual in the group will interact with all $N^{g}-1$ other individuals in the group. Here, ${\vec{n}}^{g}=(n_{1}^{g},\ldots ,n_{d}^{g})$ is the vector of types configuration in the group *g*, $n_{j}^{g}$ is the number of individuals with type *j* in the group *g*, and the group size is $N^{g}=\sum_{i=1}^{d}n_{i}^{g}\leq n$. Each individual in the group interacts with all $N^{g}-1$ other individuals in the group, and $a_{ij}$ corresponds to the elements of the payoff matrix $A=(a_{ij}{)}_{i,j=1,\ldots ,d}$ (34). The element $a_{ii}$ captures interactions between individuals of the same type, while $a_{ij}\;(i\neq j)$ describes interactions between different types. The fitness of type *j* in group *g* is determined using an exponential payoff-to-fitness mapping,


(2) $$f_{j}({\vec{n}}^{g})=e^{\beta \;\pi_{j}({\vec{n}}^{g})},$$

where *β* is the selection intensity. This mapping allows for strong selection and guarantees that fitness is always positive, even when the payoffs are negative (16, 35). For β≪1, it also allows to consider weak selection.

**Fig. 1.**
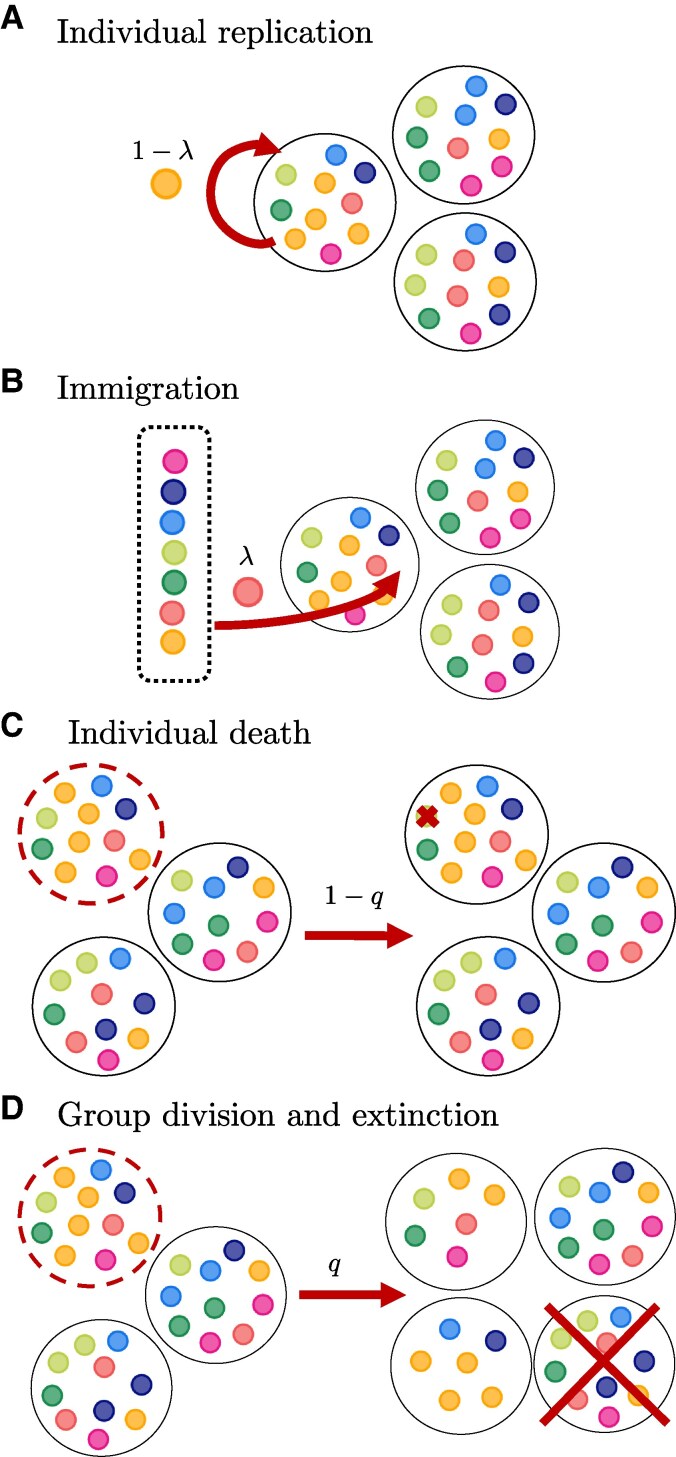
Illustration of the population dynamics processes. In this example, we consider a population structured into m=3 groups (depicted by large circles), each with a carrying capacity of n=10 individuals (small colored circles) with d=7 types (colors of individuals). The maximum total population size is N=mn=30 individuals. Individuals interact within a group through an evolutionary game, where the payoff of the game is interpreted as fitness. A) At each time step, with probability 1−λ, one individual is selected for replication proportional to its fitness, and its offspring is added to the same group. B) Alternatively, with probability *λ*, one individual immigrates from the environmental pool that contains all *d* types to one of the groups. If a group size exceeds its carrying capacity *n*, C) either one individual is removed from the group with probability 1−q, D) or with probability *q*, the group splits and its individuals are randomly distributed into two groups. To keep the number of groups *m* constant, another group is removed from the population.

The population dynamics is as follows (Fig. 1):

1. At each time step, an individual is selected to replicate with probability 1−λ proportional to their fitness, or an individual immigrates from an environmental pool to a randomly chosen group with probability *λ*. The replication probability for an individual of type *j* in a group *g* is
  (3) $$b_{\;j}({\vec{n}}^{g})=\frac{n_{j}^{g}f_{j}({\vec{n}}^{g})}{\sum_{k=1}^{d}n_{k}^{g}f_{k}({\vec{n}}^{g})}.$$
2. If the group size remains smaller than *n*, the update continues (back to step 1). If the group size exceeds *n*, one of the following occurs:
  - With probability *q*, the group splits into two and individuals are allocated with equal probability to each of the two offspring groups. If either offspring group has fewer than two individuals afterwards, the allocation process is repeated until both groups have at least two individuals. To keep the number of groups *m* constant, another group is randomly removed from the population.
  - With probability 1−q, a random individual is removed from the group.

Then, the update continues (back to step 1).

This process is iterated over many time steps, with individuals undergoing immigration, replication and death, as well as being subject to group division and extinction. Thus, the total population size *N* fluctuates between 2m and mn individuals. Figure 1 shows an illustration of this process.

As in previous work (15, 16), evolutionary dynamics is entirely driven by individual properties. Hence, groups with fitter individuals reach carrying capacity faster and may split more often. This leads to three timescales in the population: individual replication, immigration from the environmental pool, and group division. These are regulated by the immigration rate *λ* and the group-splitting probability *q*. When the splitting probability is q≪1, group-level selection is much slower than individual-level selection, as group splitting is triggered by individual replication exceeding the group carrying capacity *n*. Even when q≈1 (8), group splitting remains on a slower timescale, as it takes some time before a group is able to reach carrying capacity *n* again after splitting.

## Results

In order to investigate the effect of the group structure and to assess the effect of the group sizes on evolutionary dynamics, we simulate a population with a total maximum size of N=mn, structured into *m* groups with *n* individuals. Unless otherwise stated, we keep a total maximum population size fixed, N=120, and vary the number of groups *m* and maximum group sizes n=N/m. We limit the total population size to N=120 individuals to keep computation times reasonably short, while also allowing to explore a range of group sizes and number of groups. We focus on a case with d=1000 types, selection intensity β=1.0, and immigration rate λ=0.1. The payoff matrix *A* is sampled from a Gaussian distribution with mean μ=0 and variance $\sigma^{2}=1$, providing a wide range of interactions between multiple types (36, 37). The same realization of the payoff matrix is used across all simulation results. The immigration rate *λ* is kept large enough to promote diversity in the population. We consider two scenarios for the group splitting probability *q*: rare splitting, q=0.001, and frequent splitting, q=1.0. By adjusting the group splitting probability *q*, we can effectively control the timescale of group-level events relative to individual-level events. To compare the impact of the group structure in the dynamics, we also consider two single-group (m=1) populations: one with a maximum size N=nm, and another with a maximum size *n*, where *n* corresponds to the smallest group size considered in the group-structured population. Single-group populations do not split, q=0.0.

### Effect of group structure on abundance dynamics

**Fig. 2.**
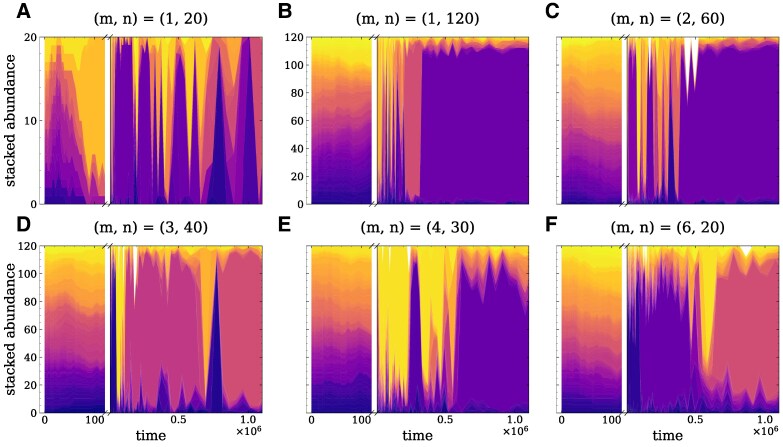
Effect of different population structures on abundance dynamics. Stacked abundances of the types present in the population are shown, with each type represented by a different color. The time axis is broken to distinguish between the initial dynamics, where there are at most N=mn types in the population, and the late dynamics. Panels compare different (m,n) population configurations: single group (q=0.0) with size A) N=20, and B) N=120, and group structured populations with size N=120 and splitting probability q=0.001, partitioned as C–F) (m,n)=(2,60), (3,40), (4,30), and (6,20). Other parameters are: immigration rate $\lambda =10^{-1}$, selection intensity β=1.0, and number of types d=1,000. The d×d payoff matrix with Gaussian distributed entries, $N(\mu =0,\sigma^{2}=1)$, was kept the same across all subplots.

We start by analyzing the time-series of the absolute abundances in the population. We compare the dynamics of a small single-group, N=20 (Fig. 2A), a large single-group, N=120 (Fig. 2B), and group structured populations with N=120 partitioned into m=2,3,4, and 6 groups (Fig. 2C–F). The maximum number of types present in the population is constrained by the group and total population size. Initially, the types present in the population exhibit the same abundance, as the initial distribution of individuals is random. Once the individual dynamics has taken place and a type has established in the population, the arrival of immigrants promote fluctuations in their abundances. Due to immigration, even if a type goes extinct due to population dynamics, it can reappear in the population.

As expected, the abundance dynamics in small single-group, (m,n)=(1,20) (Fig. 2A), is more susceptible to fluctuations than the large single-group, (m,n)=(1,120) (Fig. 2B). In the group structured populations, the turnover of the most abundant type is faster than in the single-group populations, and it increases with the number of groups *m* (Fig. S3A). Even though the turnover for the most abundant type is higher in the group-structured populations, the group-structured populations maintain a high similarity between the abundance time-series for a long time (Fig. S4A). We observe that the similarity between the abundance time-series across all population configurations is high for short-time windows and decreases as the time window increases.

In the group structure populations with low splitting probability q=0.001, groups are often at their carrying capacity and have the maximum number of individuals *n*. When q≪1, individual dynamics (replication and death events) is much faster than group-level dynamics (group division and extinction), as a group division and extinction are rare events. By structuring the large single-group population into smaller groups, we impose a smaller effective population size, such that an individual is exposed to lower local competition. As a result, when an immigrant arrives, it has a higher probability of becoming established within the group. If the group then splits, the migrant can spread throughout the population.

When q=1.0, groups split as soon as they exceed their carrying capacity *n*, so groups are often below their carrying capacity. As groups split more frequently, individual types do not have the time to fixate in a group before it splits, and the population is typically well below the total carrying capacity (Fig. S2). The turnover behavior is similar to the case with low splitting probability, but the average time as most abundant type is shorter when q=1.0 (Fig. S3B). As the number of groups increases, the similarity between the abundance time-series increases and remains higher for longer time windows when q=1.0 (Fig. S4B). Both results are expected: as groups split more often when their group size decreases, they are more likely to reach their carrying capacity faster, thus leading to a faster group-level dynamics when q=1.0, .

### Selection of interactions in the population

To characterize the selection of interactions in the population and understand the role of the group structure in this process, we first need to define the interactions between types. Instead of using an ecological classification for the interactions, eg mutualism, parasitism, and competition (38, 39), we focus on the dynamical outcome of the pairwise interactions (see Ref. (33) for a discussion on this topic). From the payoff matrix, we compute the pairwise interactions between types *i* and *j* and classify them into three categories based on their dynamical outcomes: dominance of either type *i* or type *j*, coexistence, or bistability (see SI Appendix Classification of pairwise interactions based on their stability section for details).

**Fig. 3.**
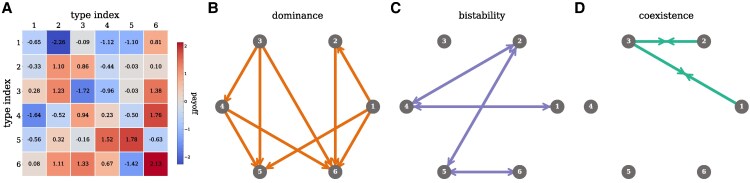
Example of the classification of pairwise interactions for a payoff matrix. A) Payoff matrix with d=6 types with Gaussian distributed entries, $N(\mu =0,\sigma^{2}=1)$. This matrix can be represented as a graph composed of *d* nodes, where each node represents a type, connected by d(d−1)/2 links corresponding to the pairwise interactions between types. Each interaction is classified according to its stability (see SI Appendix Classification of pairwise interactions based on their stability section for details), indicated by the color of the links, and for clarity, plotted only in the panel corresponding to its stability type: B) dominance (*i* dominates *j*, i←j), C) bistability (i←→j), and D) coexistence (i→←j). For example, type 2 dominates type 1, while types 5 and 6 are bistable, and types 2 and 3 coexist. For this manuscript, we focus on d=1,000.

We illustrate this classification of interactions using a payoff matrix with d=6 types (Fig. 3A) and compute the interactions between each type. To visualize these interactions, we construct a graph where each node represents a type, and each edge represents the interaction between two types. Figure 3B–D shows this visualization for each type of interaction separately, dominance (Fig. 3B), bistability (Fig. 3C), and coexistence (Fig. 3D). This classification of interactions helps us identify patterns and relationships between the types that are not immediately apparent from the payoff matrix alone. For example, a random payoff matrix would have on average 50% dominance, 25% bistability and 25% coexistence interactions. In Fig. 3, we observe that there are fewer coexistence interactions, while more dominance and bistability interactions than expected.

We compute the frequency of these interaction stability types in the payoff matrix to establish a baseline for their expected frequencies in the population. In Fig. S1, we plot the frequency of interaction stability types for the d=1,000 types used throughout our simulations. By ranking these interactions according to the types’ self-payoffs, we observe that types with higher self-payoffs tend to have more dominance (as the dominating type) and bistability interactions, whereas types with lower self-payoffs are more likely to be the dominated type and have more coexistence interactions.

**Fig. 4.**
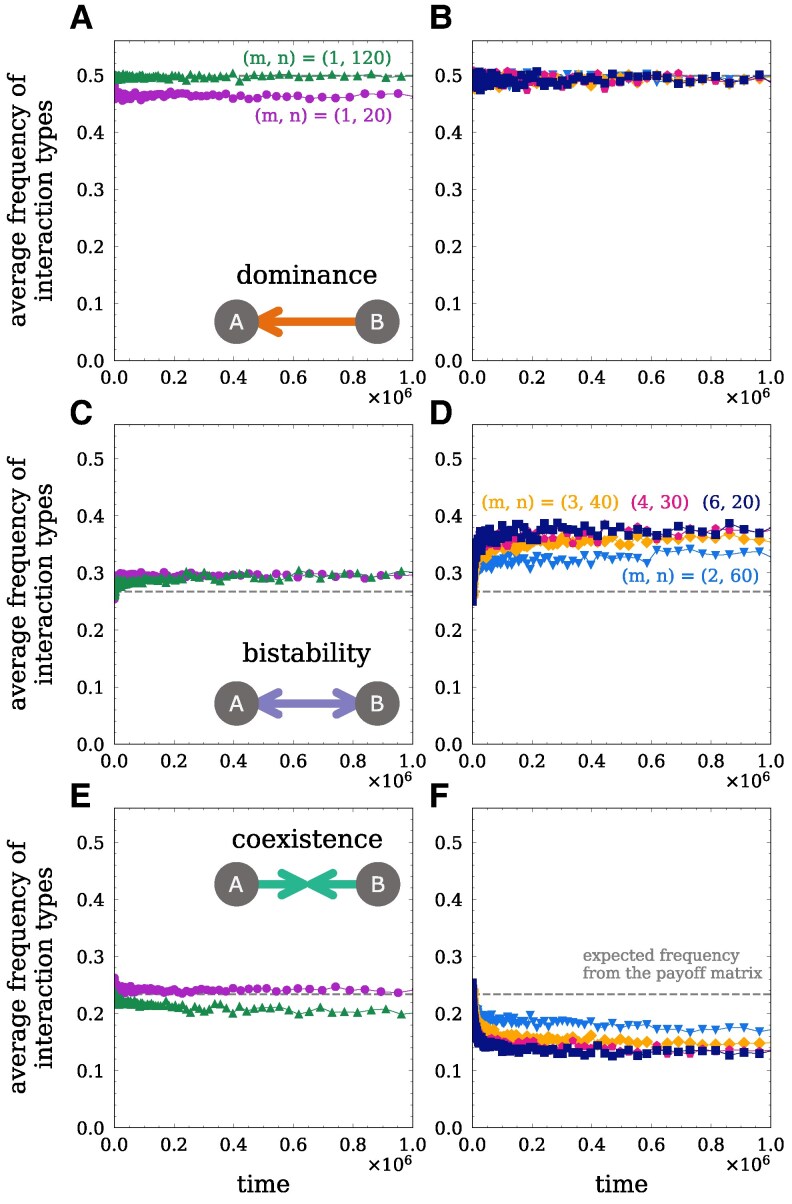
Group structure alters the frequency of interaction types. The average frequency of dominance interactions is shown on the top row, bistability on the middle row, and coexistence interactions on the bottom row. We compare the interactions between single-group populations (shown on the left) and populations with N=120 and m=2,3,4,6 groups (shown on the right). Dashed gray lines indicate the expected frequencies of stability types from the payoff matrix. However, we observe differences in the frequencies between single-group and group-structured populations. Specifically, increasing the number of groups *m* promotes bistability interactions and decreases coexistence interactions. Population configurations are as described in Fig. 2.

To analyze how population structure influences the selection of interactions, we compare the relative frequency of interaction types for the population configurations described in the previous section. Individuals only interact with other individuals in their group, so interaction types are measured between effectively interacting individuals, ie individuals that are in the same group at the same time. If group size were the sole determinant of group-structured population dynamics, one would expect the frequency of interactions to be similar between populations with the same group size, eg (m,n)=(1,20), and (6,20). Alternatively, if the maximal total population size were the sole determinant, one would expect the frequency of interactions to be similar between populations with the same total size, eg (m,n)=(1,120), (2,60), and (6,20). However, we observe that these populations have different relative frequencies of interactions (Fig. 4), namely group-structured populations exhibit an increased frequency of bistability and a decreased frequency of coexistence interactions compared to the single-group populations. These results indicate that both population size and group structure (group sizes and number of groups) influence the frequency of interactions.

To further explore the effect of the population structure on the selection of interactions, we analyze cases with fixed number of groups *m* or fixed maximum group size *n*. By keeping either the number of groups *m* or the maximum group size *n* constant, we can isolate the effects of having more groups or larger groups in the population. When increasing the number of groups *m* while keeping the maximum group size constant, n=20, we observe a decrease in the frequency of coexistence interactions and an increase in bistability interactions (Fig. S5). This effect saturates after increasing *m* by a few groups, and further increasing the number of groups does not significantly alter the frequency of interactions. Conversely, when keeping the number of groups constant, m=6, and varying the maximum group size *n*, we observe a nonmonotonic relationship between the frequency of interactions and the group size *n* (Fig. S6). For intermediate group sizes, there is an increase in the frequency of bistability interactions and a decrease in the frequency of coexistence interactions. Further increasing the group size leads to a decrease in the frequency of bistability and an increase in the frequency of coexistence interactions, approaching the frequencies observed in the small single-group population. These results show that increasing the number of groups *m* or the maximum group size *n* may have contrasting effects on the selection of interactions in the population.

We also examine the impact of the splitting probability *q* on the selection of interactions by analyzing the case when q=1.0. In this case, dynamics are driven solely by replication of individuals and group-level events, as there is no death of individuals. Fig. S7 shows that the selection of interactions with q=1.0 follows a similar pattern as for q≪1 (Fig. 4), with the frequency of coexistence interactions decreasing and bistability interactions increasing with the number of groups *m*. In the next section, we will further explore the relationship between the selection of interactions and the abundance of types in the population.

### Self-payoff correlates with relative abundance

**Fig. 5.**
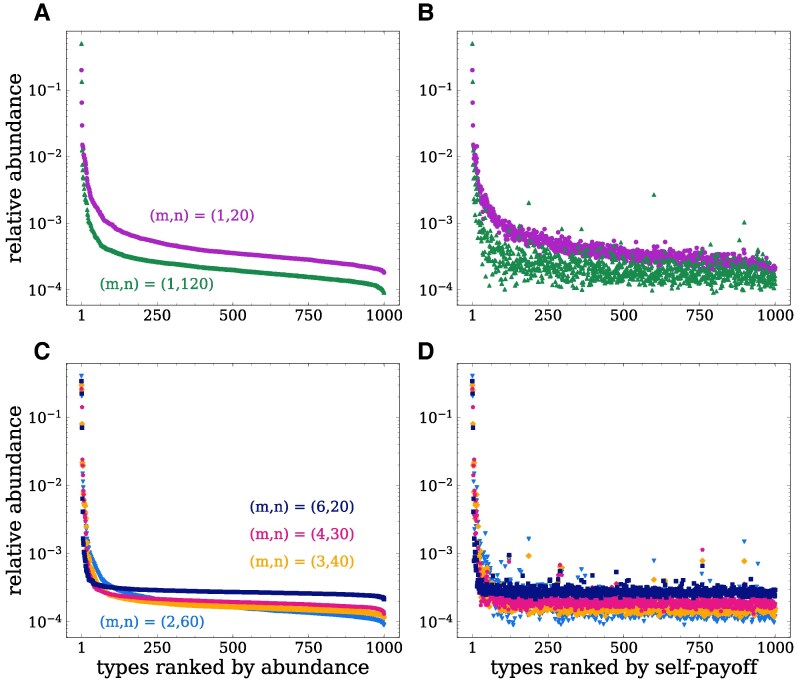
Distribution of ranked average relative abundances depends on population structure. Average relative abundance time-averaged in the long-run (using the last 15 time points of the simulation) ranked by the types’ relative abundance (left), and by their self-payoff (right). We compare the distributions between single-group populations (top row) and populations with *N* = 120 and *m* = 2, 3, 4, 6 groups (bottom row). Single-group populations exhibit a more even distribution of abundances across ranks compared to group-structured populations. Group-structured populations tend to have a few very abundant types and more types with lower abundances, which gets more pronounced as the number of groups *m* increases. Population configurations are as described in Fig. 2.

In the previous section, we have seen that population structure alters which interactions are selected depend on the population structure. To explore how these results relate to the different observed abundance patterns (Fig. 2 and Fig. S2), we analyze how the average relative abundance of types changes in the long-run depending on the population structure. We rank the types in two different ways (Fig. 5): according to their average abundance and their self-payoff. Both rankings are correlated, indicating that types with higher self-payoff are also more abundant in the population (Fig. S8).

All large populations (N=120) exhibit an increased abundance of the most abundant type compared to the small population (N=20) (Fig. 5). This most abundant type is also the type with the highest self-payoff. As seen in the previous section, types with higher self-payoff have a higher frequency of dominance (as the dominant type) and bistability interactions, while types with lower self-payoff have higher frequency of dominance (as the dominated type) coexistence interactions (Fig. S1). When groups are mostly homogeneous, having a higher self-payoff increases the chance of a type spreading through the population, leading to a higher abundance of types with higher self-payoff in the large single-group and group-structured populations (Fig. 5). The increased abundance of these types with high self-payoff is responsible for the higher frequency of dominance interactions at the cost of lower frequency of coexistence interactions in large populations (Fig. 4). In contrast, in the small single-group population, the relative abundance of the most abundant types is lower than in the large single-group population, but the relative abundances of the other types are higher than in the large single-group. This leads to the higher frequency of coexistence interactions and lower frequency of dominance interactions in the small single-group population (Fig. 4A and E).

Focusing on the group-structured populations, we observe that these populations typically have only a few very abundant types. As *m* increases, types with higher self-payoff become more abundant, while the other types become equally distributed at lower abundances (Fig. 5C and D). This ultimately leads to an increase in the frequency of bistability interactions in the group structured populations, at the cost of lower frequency of coexistence interactions. A similar behavior is observed when the maximum group size *n* is constant, n=20, and the number of groups *m* is increased (Fig. S10). The abundance of types with highest self-payoff increases with *m*, while all other types have their abundances concentrated at lower abundances. This effect saturates after increasing *m* by a few groups, explaining the saturation in the increase of bistability and the decrease of coexistence interactions (Fig. S5). In the case where the number of groups is fixed, m=6, we observe a maximum in the abundance of the most abundant type with *n* (Fig. S9). Due to β=1.0, selection is already very effective in small groups, but stochastic effects arise in small groups from (i) incoming migrants that immediately have a high relative abundance in a small groups and (ii) the stochastic effect arising from nonhomogeneous groups that are splitting. In Fig. S9, this is reflected in the very low abundance that migrants have in larger groups. As the group size *n* increases, the abundance distribution becomes more concentrated in middle and lower abundances, and there is a lower correlation between frequency of types and self-payoff. These results are consistent with the observed nonmonotonic relationship between the frequency of interactions and increasing group size (Fig. S6). By keeping the total population size fixed and increasing the number of groups *m* while decreasing maximum group sizes *n*, we observe a combination of these two effects. As the number of groups *m* increases, types with lower self-payoff are concentrated at similar lower abundances, while the types with the highest self-payoff remain at high abundances. As the group size *n* decreases, the correlation between the types frequency and their self-payoff increases. These effects together lead to the increase in the frequency of bistability and the decrease in the frequency of coexistence interactions observed in the group-structured populations (Fig. 4).

In the high splitting probability case, q=1.0, higher-ranked types are selected through the fast assortment dynamics and are highly abundant. Lower-ranked types typically do not have enough time to fixate in a group, as groups split as soon as they exceed their carrying capacity. As a result, their abundance is lower than in the q=0.001 case and decreases further as group sizes decrease (Fig. S11 bottom). Ultimately, this leads to a higher frequency of bistability interactions in the group structured populations with q=1.0 compared to the q=0.001 case (Fig. S7).

### Group structure leads to higher diversity under low splitting probability

In the previous sections, we analyzed the selection of interactions in group-structured populations. Let us now turn to how the group structure affects the diversity of these populations. To assess the effects of group structure on community composition and diversity, we analyze different diversity indices, such as richness and the Shannon diversity index (40). We examine these indices at both the population and group levels and also consider the similarity between groups (see more details in SI Appendix Materials and methods section).

**Fig. 6.**
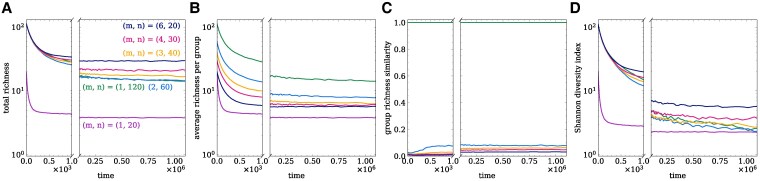
Group structure leads to higher diversity indices for low splitting probability. We show the diversity metrics: A) total richness, B) average richness per group, C) group richness similarity, and D) total Shannon diversity index. Population configurations are: single-group populations with size (m,n)=(1,20) and (1,120), and group structured populations with N=120 and splitting probability q=0.001, partitioned as (m,n)=(2,60), (3,40), (4,30), and (6,20). Increasing the number of groups *m* leads to higher total richness, total Shannon diversity, while decreasing group richness similarity. When comparing groups with similar size, average group richness is higher in group-structured than in single-group populations. The description of each diversity index is given in SI Appendix Materials and methods section. Population configurations are as described in Fig. 2.

At the population level, group-structured populations (N=120) have higher richness compared to the large single-group population, (m,n)=(1,120) (Fig. 6A). Even at the group level, group-structured populations have higher group richness in the long-run than their single-group counterparts (eg comparing (m,n)=(1,20) and (6,20) in Fig. 6B). In single-group populations, the group and population level measures are the same (Fig. 6A and B). When examining group richness, we observe that as the number of groups *m* increases, the groups become less similar to each other (Fig. 6C). Although the initial distribution of individuals is random, after the dynamics has taken place, groups are not very similar to each other. The group richness similarity depends both on group size and number of groups. Larger groups tend to be more similar, whereas smaller groups differ more. Likewise, similarity is more likely to be higher when there are fewer groups and lower when there are more groups. Thus, the high richness at the population level is driven by the different composition of the groups. This higher diversity holds even if we look at other diversity indices, such as the Shannon diversity index (Fig. 6D). The Shannon diversity index differs from richness in its sensitivity to the abundance of common and rare types. Richness is more sensitive to rare types, while the Shannon index accounts for both common and rare types.

However, when splitting probability is high, q=1.0, group-structured populations exhibit lower diversity at both the population and group levels (Fig. S12). At the population level, group-structured populations have lower richness than a similar sized population, (m,n)=(1,120) (Fig. S12A), and also lower richness compared to the case with low *q*. Additionally, all group-structured populations have similar total richness, contrary to the case with low *q*. This lower diversity also holds at the group level, where group-structured populations have lower richness than the single-group population (eg (m,n)=(1,20) and (6,20), Fig. S12B). Interestingly, throughout the dynamics, these groups reach a peak in their similarity (peak in Fig. S12C) and then reach a lower similarity between groups. The dynamics driven by group splitting promote groups that are more similar to each other, with groups with a higher similarity when compared to the low splitting case, q=0.001. Similar findings hold for the Shannon diversity index: overall the group-structured populations have a lower Shannon index than the single-group populations. Even though a high splitting probability did not change the type of interactions being selected in the population, these results indicate that the timescale between individual-level and group-level events have an important role in shaping the diversity of these populations.

Similar results also hold for the frequency of homogeneous groups in the population (Fig. S13). A group is considered homogeneous if all individuals in the group are of the same type. The frequency of homogeneous groups decreases as the group size *n* increases, but increases with the number of groups *m* (Fig. S13). Similar to the frequency of interactions, when keeping *n* constant, the frequency of homogeneous groups increases with *m* up to a point, after which it saturates. We find that not only are groups more similar to each other when splitting probability is high, but they also tend to be more homogeneous (Fig. S13).

## Discussion and conclusion

Our work was motivated by the goal of understanding the role of multilevel selection on shaping interactions within host-associated microbial communities. Previous studies have focused on the evolution of cooperation and the conditions under which cooperation can be maintained (15, 19, 21). However, natural microbial communities often consist of multiple types of individuals that interact with each other in complex ways—and a type may be a cooperator in one interaction, but a defector in another one. Here, we aimed to move the discussion of multilevel selection beyond mechanisms to promote cooperation, as even the definition of cooperation in this context is not straightforward. This raised the question of how to classify interactions when there are multiple types of individuals in a population, and how multilevel selection shapes these interactions.

We proposed and studied a model for the evolutionary dynamics of multilevel selection in a multitype population. Specifically, we consider a population with *d* types of individuals and examine the impact of multilevel selection on the selection of interactions. Our motivation to consider d≫2 is 2-fold: First, to study the case where the number of types is much larger than two. Second, to ensure that the different types of interactions are present in the payoff matrix and, subsequently, in the population. By considering a large number of types, a wide range of interactions can be sampled, and our results are not sensitive to the particular realization of the payoff matrix. Instead of relying on interactions based on ecological classifications, we classified interactions based on the dynamical outcome of the pairwise interactions. This allowed us to categorize interactions as: dominance of either type 1 or type 2, bistability, or coexistence.

We considered immigration from the environment to allow extinct types to reappear in the population. This allowed us to shift the focus from analyzing fixation probabilities and extinction patterns to examining how interactions are selected depending on the population structure. If the immigration rate *λ* is too low, λ≪1, the dynamics will lead to extinction events in the population. If *λ* is high, λ=1.0, the population will reflect the composition of the environmental pool. Our choice of λ=0.1 was motivated by the need to promote diversity in the population. This allows interactions between more than one type, while also allowing for the dynamics to be driven by selection (birth) and not only immigration (λ∼1.0).

We considered an intermediate selection strength, β=1.0, to ensure that the dynamics is driven by the fitness differences between types, and not by random drift. If β≪1, the dynamics is under a weak selection regime, where differences in the payoffs do not have a large impact on the fitness of an individual. If β≫1, the dynamics is under a strong selection regime, where even very small differences in individual payoffs lead to large differences in fitness. We thus chose an intermediate value, β=1.0, as a selection strength where the differences in payoffs already have an impact on individuals’ fitness.

Our results showed that population structure, group size, and number of groups played a crucial role in shaping the frequency of each interaction type. We found that the frequency of dominance interactions increases with population size, while the frequency of coexistence interactions decreases. Furthermore, structuring a population into groups, even while keeping the total population size constant, led to an increase in the frequency of bistability interactions and a decrease in the frequency of coexistence interactions. This effect was particularly pronounced when the splitting probability was high, q=1.0. These results are consistent with previous findings of frequent bistability interactions between bacterial strains (41).

Despite the decrease in coexistence interactions, group-structured populations under low splitting probability exhibited higher diversity than single-group populations. It is worth noting that in bistability interactions, both types can potentially exclude each other depending on their initial frequencies. Thus, as soon as we have multiple groups, different types can colonize different groups, leading to a higher overall diversity. High splitting probability, however, led to lower diversity compared to low splitting probability and to single-group populations. Despite the increase in bistability interactions, the dynamics driven by frequent group splitting promoted groups that were more similar to each other and more homogeneous. These findings highlight the importance of population structure and the timescale of group-level events in shaping interactions and population composition, particularly when considering multitype communities.

By considering more than two types d>2 in the population, we revealed a more complex view on multilevel selection than a mechanism to promote cooperation. We argued that the focus should shift from identifying cooperative interactions in multitype populations, which is inherently complex and potentially misleading, to understanding how it leads to different context-dependent strategies to maximize fitness in different groups. Multilevel selection has the potential to lead to different outcomes depending on the population structure and the interactions between types.

Our model has several assumptions that warrant discussion. We assumed group splitting to be triggered by individual replication, which is a simplification of the group-level dynamics. Exploring alternative mechanisms for group splitting, such as different modes of fragmentation, could provide further insights on the effects of group-level processes on population dynamics (42). Additionally, we picked a group randomly to be removed from the population. Our model focuses on selection factors at birth, but ignores selection at death. This is a common modeling convention in the field, and it allows us to focus on the specific effects of multilevel selection on a multitype population. Our model considered immigration from the environment and vertical transmission between groups. However, other methods of microbiome transmission, such as horizontal transmission (17, 19, 42, 43), are also known to influence the dynamics of microbial communities. In our model, horizontal transmission corresponds to migration between groups. When horizontal transmission is much stronger than vertical transmission, the group-structured population becomes similar to the well-mixed case, where all groups have a similar composition. It is also known that, in the absence of selection, microbial inheritance may shape microbiome composition (44). Further research is needed to understand how these different transmission methods affect the dynamics of the population and the selection of interactions under selection. Furthermore, we assumed pairwise interactions between types, but higher-order interactions could capture other aspects of the dynamics and provide a more comprehensive view of ecological dynamics (45–48). Finally, we assumed that all types are present in the environmental population with the same frequency, whereas the coevolution of the environment and the population can affect population dynamics and evolutionary trajectories. Future work could explore how feedback between the environment and the population influences the dynamics of multilevel selection and shapes the long-term evolution of interactions.

## Supplementary Material

pgag180_Supplementary_Data

## Data Availability

All simulations were performed using Python 3.9. The code (49) and corresponding data (50) are available on Zenodo at https://doi.org/10.5281/zenodo.17880820 and https://doi.org/10.5281/zenodo.17880247.
