# Supplementary material for: Multilevel selection in multitype populations
Source: PNAS Nexus. 2026 May 20;5(6):pgag180. doi: 10.1093/pnasnexus/pgag180 (PMC13232747; doi:10.1093/pnasnexus/pgag180)
Supplement: pgag180_Supplementary_Data [file pgag180_supplementary_data.pdf]

Multilevel selection in multi-type populations
Supplementary Information

Amanda de Azevedo-Lopes[*1] and Arne Traulsen[†1]

[1]Department of Theoretical Biology, Max Planck Institute for Evolutionary Biology, 24306 Plön, Germany

(Dated: May 18, 2026)

Contents

A Materials and Methods 1

A.1 Classification of pairwise interactions based on their stability 2

B Model implementation 3

C Supplementary figures 4

A Materials and Methods

The number of individuals with type j in the group g at time t is $n_j^g(t)$, and the vector of types configuration in the group g at time t is $\vec{n}^g(t) = (n_1^g(t), \ldots, n_d^g(t))$. The number of individuals per group, N^g, is given by

$$N^g(t) = \sum_{k=1}^{d} n_k^g(t)\,, \tag{S1}$$

which is constrained by the maximum group size n, $N^g(t) \leq n$. Thus, the total population size, $N(t)$, is

$$N(t) = \sum_{g=1}^{m} N^g(t) \leq mn\,. \tag{S2}$$

We also compute the relative abundance of type k per group g, $p_k^g(t)$,

$$p_k^g(t) = \frac{n_k^g(t)}{\sum_l n_l^g(t)}\,, \tag{S3}$$

and the total relative abundance of type k, $p_k(t)$,

$$p_k(t) = \frac{\sum_g n_k^g(t)}{\sum_{l,g} n_l^g(t)}\,. \tag{S4}$$

From the total relative abundance, we can compute different diversity indices, which are described by their Hill numbers [1],

$${}^kD(t) = \left(\sum_{i=1}^{d} p_i(t)^k\right)^{1/(1-k)}\,. \tag{S5}$$

The exponent and superscript k is the order of the diversity index, which determines the sensitivity of the index to the relative abundance of types. For example, richness corresponds to $k = 0$, which just counts the presence of types without weighing their relative abundance,

$$R(t) = \sum_{k=1}^{d} 1 - \delta_{0,p_k(t)}\,. \tag{S6}$$

*azevedo@evolbio.mpg.de

†traulsen@evolbio.mpg.de

The Shannon diversity index corresponds to $k = 1$,

$$H(t) = \exp\left(-\sum_{k=1}^{d} p_k(t) \ln p_k(t)\right) , \quad \text{(S7)}$$

and the inverse Simpson diversity index corresponds to $k = 2$,

$$S(t) = \frac{1}{\sum_{k=1}^{d} p_k(t)^2} . \quad \text{(S8)}$$

These three indices calculate the average diversity of the population, but using different types of means: arithmetic, geometric, and harmonic [2]. Richness, R, corresponds to the number of types in the whole population without taking their respective abundances into account, thus being very sensitive to rare types [1]. The Shannon diversity index, H, is equivalent to weighing all types by their frequency, without favoring either common or rare types [1]. The inverse Simpson diversity index, S, also weighs types by their frequency, but it is more sensitive if some types are much more abundant than others [3]. These diversity indices may be measured considering different levels of the population, such as considering the population as a whole, at the group level, and between groups [4]. For example, richness at the group level becomes the average group richness, $R_\alpha(t)$,

$$R_\alpha(t) = \frac{1}{m} \sum_{g=1}^{m} \sum_{k=1}^{d} \left(1 - \delta_{0,p_k^g(t)}\right) , \quad \text{(S9)}$$

The between groups diversity index can be measured by taking the ratio between the population diversity and average group diversity, e.g., $R_\beta(t) = R(t)/R_\alpha(t)$. It will be minimal when all groups are equal, and maximal when all groups are different, equal to the number of groups m. From the diversity between groups, it is possible to measure the similarity between them. For the species richness, the similarity between groups is given by

$$R_G(t) = \frac{1/R_\beta(t) - 1/m}{1 - 1/m} . \quad \text{(S10)}$$

The richness similarity is zero when all m groups are completely distinct from each other, and is equal to one when all m groups are identical in species composition.

We also compute the Bray-Curtis similarity between abundance time series of the population at different time points, t_1 and t_2. It is defined as

$$\mathrm{BC}(\vec{n}(t_1), \vec{n}(t_2)) = \sum_{i=1}^{d} \frac{\min(n_i(t_1), n_i(t_2))}{\mathrm{mean}(n_i(t_1), n_i(t_2))} , \quad \text{(S11)}$$

where $\vec{n}(t_1)$ and $\vec{n}(t_2)$ are abundances of the population at times t_1 and t_2, and $n_i(t_1)$ and $n_i(t_2)$ are the abundances of type i at times t_1 and t_2. If the abundances at t_1 and t_2 are identical, i.e., $\vec{n}(t_1) = \vec{n}(t_2)$, then $\mathrm{BC}(\vec{n}(t_1), \vec{n}(t_2)) = 1$. If they have no types in common, then $\mathrm{BC}(\vec{n}(t_1), \vec{n}(t_2)) = 0$. We compute the Bray-Curtis similarity for a distance τ between time points,

$$C(\tau) = \langle \mathrm{BC}\left(\vec{n}(t), \vec{n}(t+\tau)\right) \rangle_t , \quad \text{(S12)}$$

and we average it over time t and over different samples.

A.1 Classification of pairwise interactions based on their stability

From a payoff matrix A with d types, we determine the stability of the $d(d-1)/2$ pairwise interactions among the d different types. We use this classification to construct an interaction network (main text, Fig. 3). Let us consider two types, i and j and determine the stability between them. In a large population, the dynamics of their abundances x_i and x_j is captured by the replicator equation

$$\begin{aligned} \dot{x}_i &= x_i \left[f_i - \bar{f}\right] \\ \dot{x}_j &= x_j \left[f_j - \bar{f}\right] , \end{aligned} \quad \text{(S13)}$$

where f_i is the fitness of type i, which depends on the matrix entries and the abundance of each type in the population. The average population fitness, $\bar{f}$, is given by

$$\bar{f} = x_i f_i + x_j f_j . \quad \text{(S14)}$$

Considering that $x_i + x_j = 1$, we can introduce a variable $x = x_i$ and rewrite Eqs. (S13) into

$$\dot{x} = x(1-x)\left[f_i(x) - f_j(x)\right] . \tag{S15}$$

From Eq. (S15), we can determine the stability between the type i and j. There are four possible dynamical outcomes of these interactions: dominance of either type i or type j, bistability, or coexistence. In dominance interactions only one type is stable: either type i dominates type j, or vice-versa, i.e., $x = 0$ or $x = 1$. If $f_i(x) > f_j(x)$ for all x, type i dominates, and the only stable fixed point is $x = 1$. Conversely, if $f_i(x) < f_j(x)$ for all x, type j dominates and $x = 0$ is the stable fixed point. In coexistence interactions neither pure state ($x = 0, 1$) is stable. Instead, the population converges to a stable fixed point $x \in (0, 1)$, where both types coexist. Bistability occurs when both pure states are stable, with an unstable fixed point between them. In this case, the population can converge to either one of them, $x = 0$ or $x = 1$ depending on the initial conditions.

Throughout our simulations, we consider a payoff matrix A in which all entries are drawn independently from a standard normal distribution, $\mathcal{N}(\mu = 0, \sigma^2 = 1)$. For this distribution, the probability of observing a dominance interaction is 0.5, while the probabilities for bistability or coexistence interactions are each 0.25. To illustrate these interactions, Fig. 3 shows an example payoff matrix for 10 types and its corresponding stability graph. Although the payoff matrix in Fig. 3a corresponds to a complete graph among the types, we decompose it into three separate graphs based on the interaction types (Fig. 3b-d). In this graph, each node represents a type, and each edge between nodes indicate the stability of the corresponding pairwise interaction. For dominance interactions, e.g., type i dominates type j, the edge is directed toward the dominant type, $i \leftarrow j$. For bistability interactions, edges are bidirectional, $i \longleftrightarrow j$. For coexistence interactions, links are represented with opposed arrows, $i \rightarrow\leftarrow j$.

The frequencies of these interaction types can be modulated by the types self-payoff (i.e., the diagonal entries of the payoff matrix). By changing the self-interactions, one can change the proportion of the various stability interactions. In Fig. S1, we plot the proportion of the interaction frequencies for a matrix with $d = 1000$ types, ranking the types according to their self-payoffs. We observe that types with higher self-payoffs tend to have a higher proportion of dominance (being the dominant one) and bistability interactions, while types with lower self-payoffs tend to have a higher proportion of dominance (being the dominated one) and coexistence interactions.

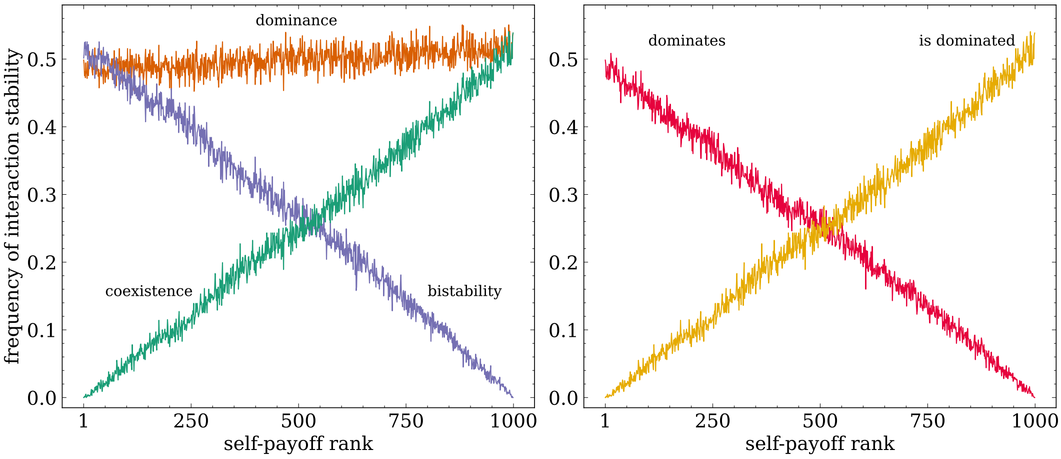

Figure S1 Frequency of stability interactions as a function of the ranked self-payoff. We classify the pairwise interactions based on their stability for each type in a payoff matrix with $d = 1000$ types and Gaussian distributed entries, $\mathcal{N}(\mu = 0, \sigma^2 = 1)$. (left) For each focal type, ranked by their self-payoff, we plot the fraction of each interaction type: orange indicates dominance, purple indicates bistability, and green indicates coexistence. (right) Dominance interactions are further divided into two categories: which type dominates the other (red), and which type is dominated (yellow). We observe that high-ranked types predominantly have dominance (as the dominant type) and bistability interactions, whereas the low-ranked types predominantly have dominance (as the dominated type) and coexistence interactions.

B Model implementation

Simulations were implemented in Python. The code used in the simulations and to generate the figures is available on Zenodo at `https://doi.org/10.5281/zenodo.17880820`. The data files used to generate the figures have been deposited at `https://doi.org/10.5281/zenodo.17880247`. All simulations were initialized in a random state,

where all m groups are populated with n individuals with their types randomly chosen between the d possible ones. Number of types is $d = 1000$, immigration rate λ from the environment is $\lambda = 10^{-1}$. Types payoffs are drawn from a normal distribution with $\mu = 0$ and $\sigma = 1$. We consider two scenarios of group splitting: rare splitting $q = 0.001$, and frequent splitting $q = 1.0$. The population evolves through a frequency-dependent Moran process, and its size may vary between $2m$ and nm individuals as groups split. Averages were taken over at least 1000 replicates (different realizations of initial conditions and stochasticity).

C Supplementary figures

As described in the main text, unless otherwise stated, reference parameters in simulations are: immigration rate $\lambda = 10^{-1}$, selection intensity $\beta = 1.0$, number of types $d = 1000$, and $d \times d$ payoff matrix with Gaussian distributed entries, $\mathcal{N}(\mu = 0, \sigma^2 = 1)$.

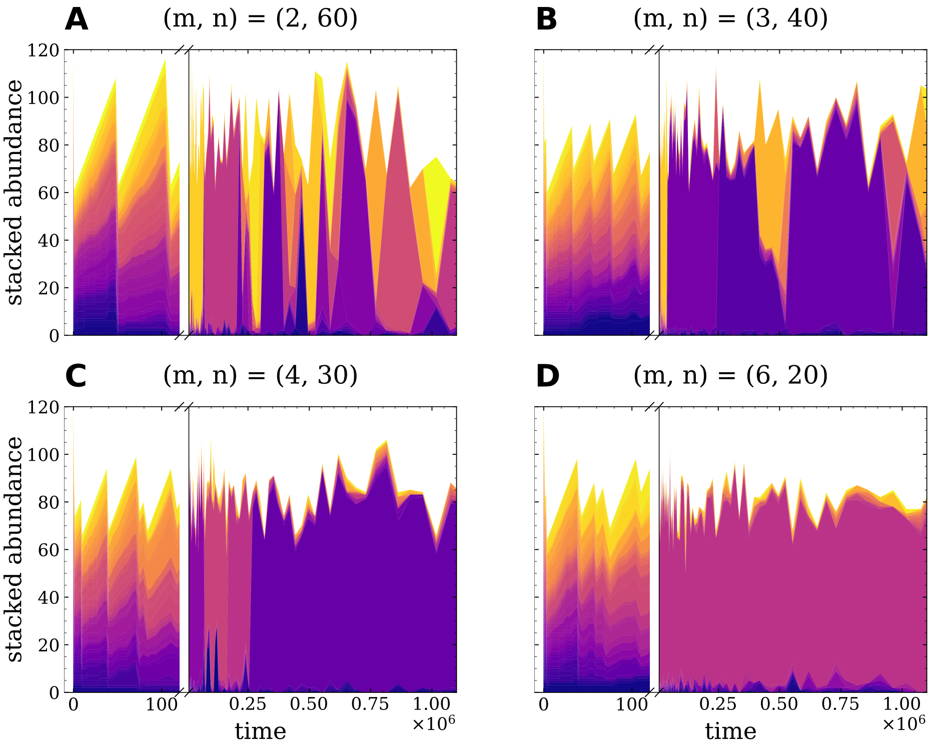

Figure S2 Effect of high splitting probability, $q = 1.0$**, on abundance dynamics.** Stacked abundances of the types present in the population are shown, with each type is represented by a different color. Panels **A-D** compare group structured populations with size $N = 120$ partitioned as $(m, n) = (2, 60)$, $(3, 40)$, $(4, 30)$, and $(6, 20)$. Initially, all groups are at carrying capacity and up to $N = mn$ types can be present in the population. Because groups split whenever they would exceed their carrying capacity, the population remains consistently below the total population size N.

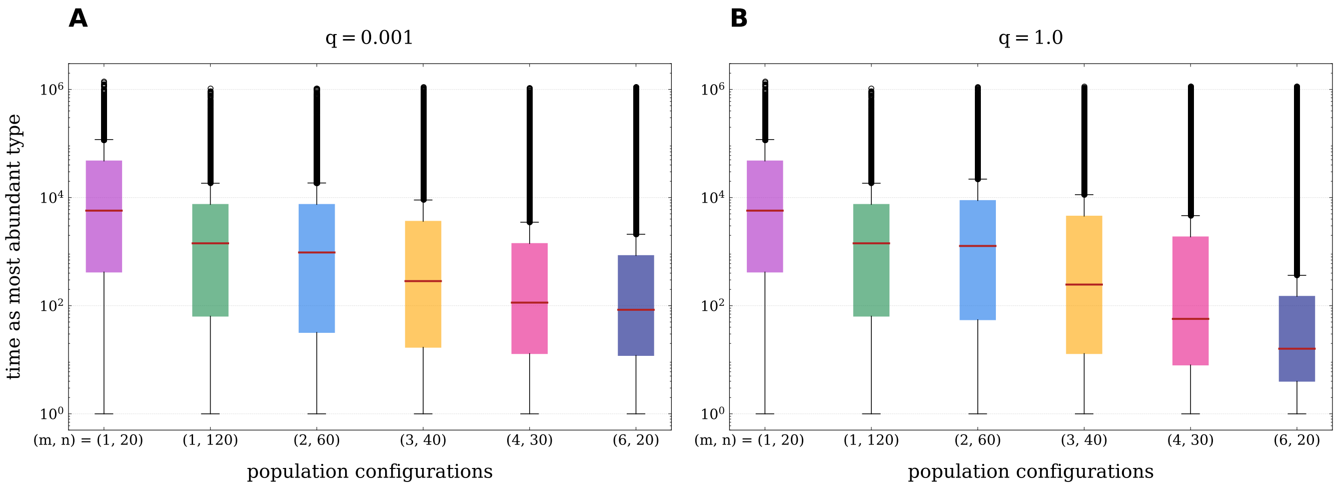

Figure S3 Turnover time for the dominant type is higher as number of groups m increases. We compute the time each type stays as the most abundant type in the population for each population configuration on a logarithmic scale. **A** shows the group structured populations with low splitting probability, $q = 0.001$, while **B** shows the group structured populations with high splitting probability, $q = 1.0$. The median time is indicated by a red line, and the box indicates the region between the first and third quartiles. Even though all population configurations have a wide distribution of times and similar extreme values (indicated by black circles), the median time decreases as the number of groups m increases (notice the logarithmic scale on the time axis). Population configurations are as described in Fig. 2 in the main text for panel A and Fig. S2 for panel B.

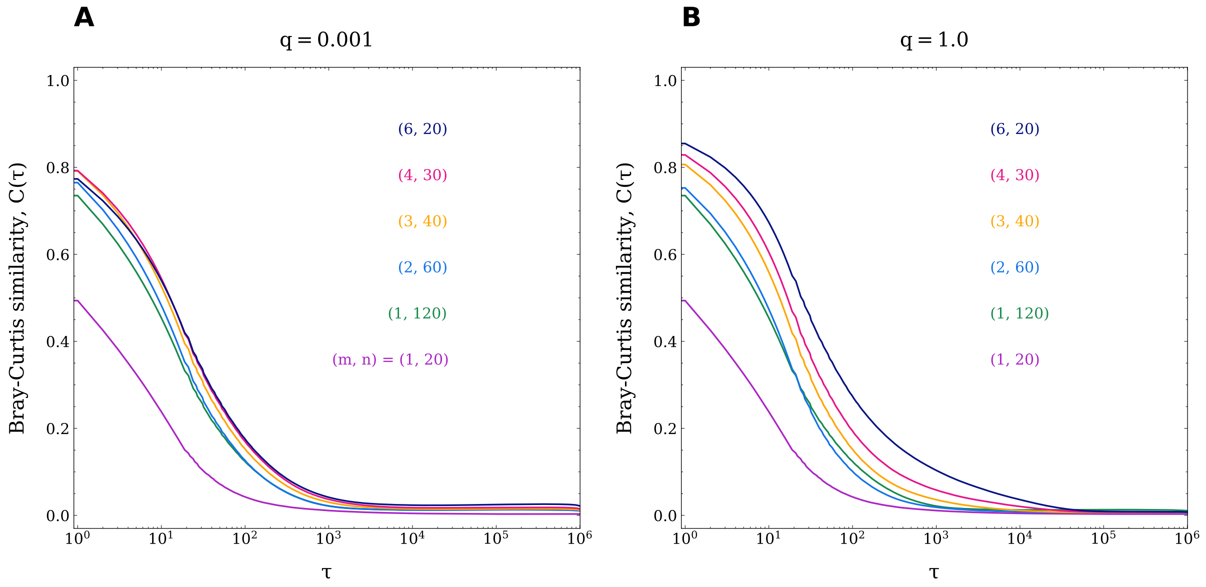

Figure S4 Decay of Bray-Curtis similarity for a fixed distance τ between two time points. We compute the Bray-Curtis similarity between the abundance profiles of the population at two time points t_1 and t_2 separated by a fixed distance $\tau = |t_1 - t_2|$ for the different (m, n) population configurations: single group ($q = 0.0$) with size $N = 20$ and 120, and multiple groups with total size $N = 120$ and splitting probability **A** $q = 0.001$ and **B** $q = 1.0$. Similarity across all population configurations is high for short time windows and decreases as the time window increases. **A** Similarity is higher for the large populations. As perhaps expected, the small single group has a lower similarity, as it is more susceptible to fluctuations and arrival of new types in the population. **B** When $q = 1.0$, similarity is higher when compared to the low splitting probability case, and as the number of groups increases, the similarity in the population also increases. Population configurations are as described in Fig. 2 in the main text for panel A and Fig. S2 for panel B.

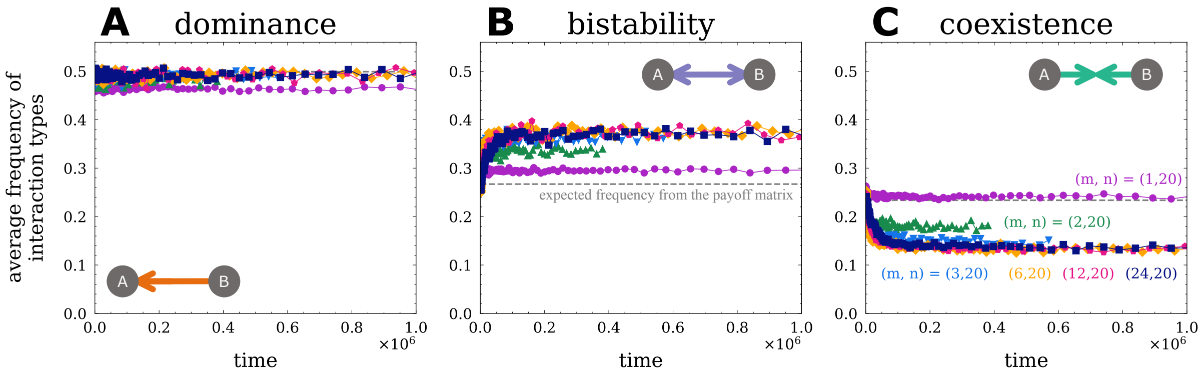

Figure S5 Further increasing the number of groups m does not affect frequency of stability types with a fixed maximum group size $n = 20$. Average frequency of **A** dominance, **B** bistability, and **C** coexistence interactions for constant group size $n = 20$ while varying the number of groups $m = 1, ..., 24$. The frequency of dominance interactions is increased when comparing $m > 1$ to $m = 1$ populations. The frequency of bistability interactions increases with the number of groups, while the frequency of coexistence interactions decreases. The effect of increasing the number of groups saturates quickly, and further increasing the number of groups does not significantly alter the frequency of interactions.

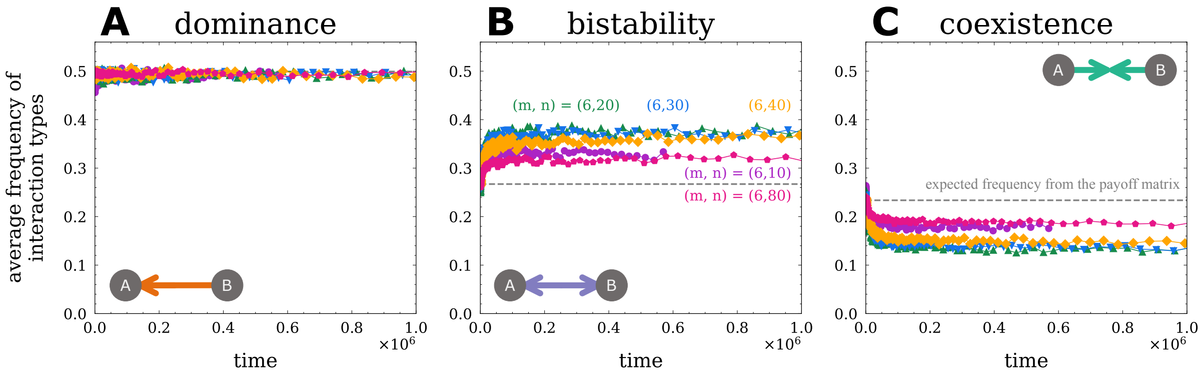

Figure S6 Non-monotonic behavior of the frequency of stability types with increasing group size n, while keeping number of groups constant, $m = 6$. Average frequency of **A** dominance, **B** bistability, and **C** coexistence interactions for a fixed number of groups $m = 6$ while varying the group sizes $n = 10, ..., 80$. The frequency of bistability and coexistence interactions display a non-monotonic behavior when increasing the group size n. Notably, the frequency of dominance interactions is not significantly affected by the group size n when $m > 1$.

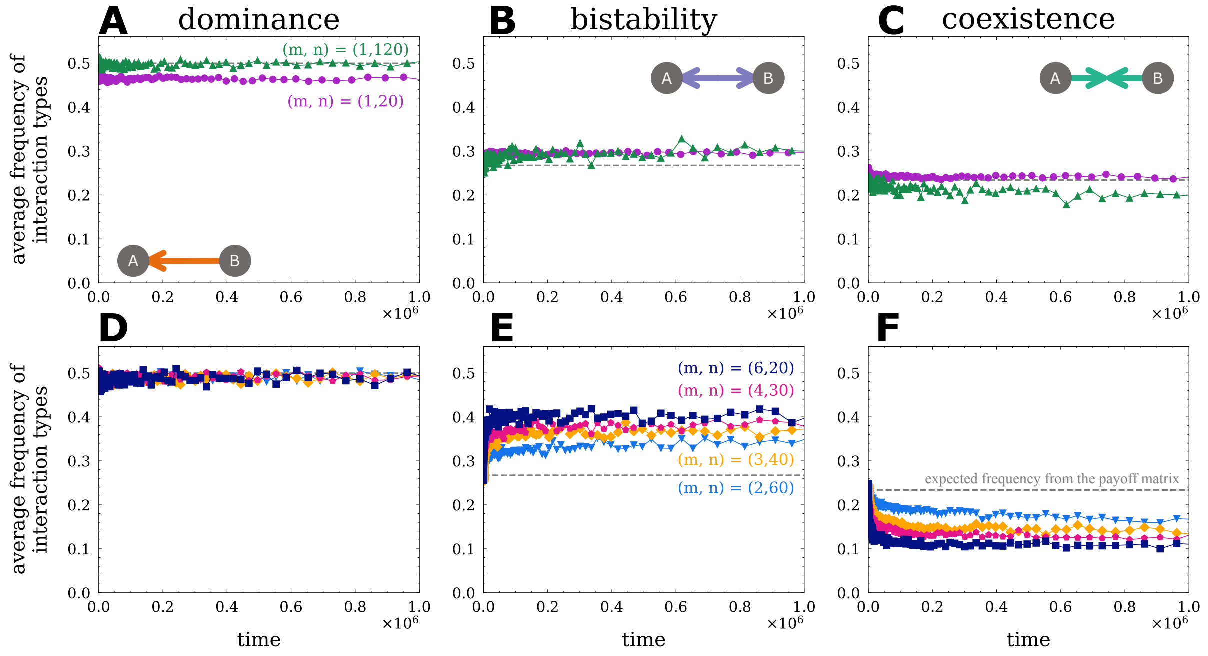

Figure S7 High splitting probability $q = 1.0$ promotes a higher frequency of bistability and lower frequency of coexistence interactions. The frequency of dominance interactions is shown on the left column, bistability on the middle column, and coexistence interactions on the right column. Single-group populations are shown on the top row (same data from main text, Fig. 4), while populations with $N = 120$ and $m = 2, 3, 4, 6$ groups are shown on the bottom row. Dashed grey lines indicate the expected frequencies of stability types from the payoff matrix. Increasing the number of groups m increases the fraction of bistability and decreases the fraction of coexistence interactions. High splitting probability increases this effect when compared to the low splitting case.

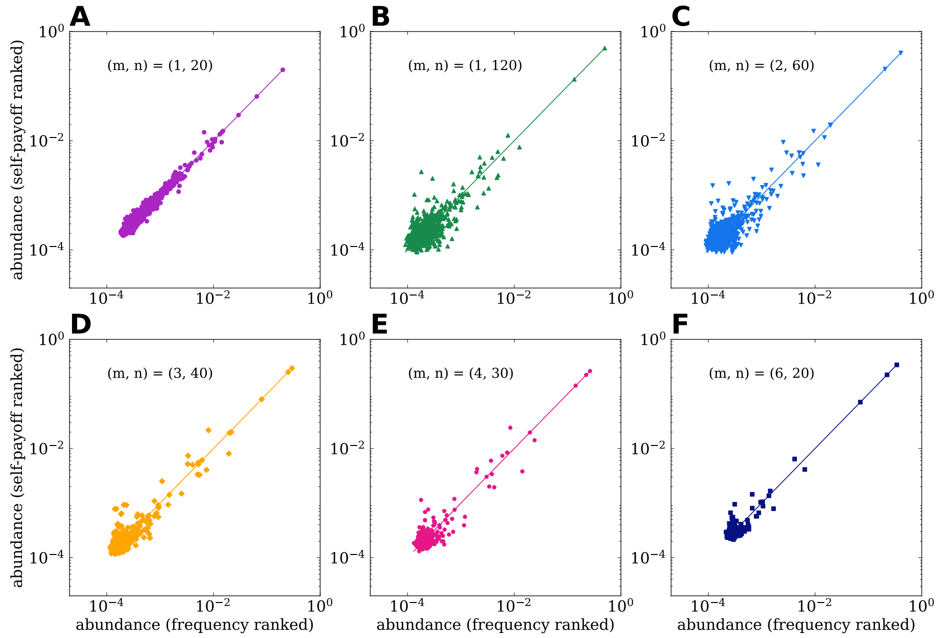

Figure S8 Types with higher self-payoff tend to be more abundant across different population configurations. Each panel shows the average abundance of types ranked against both their average abundance and their self-payoff for populations with varying number of groups m and maximum group sizes n. The strong correlation observed between both rankings indicates that self-payoff is a key determinant of type abundance. Averages were taken over the last 15 measurements of the dynamics.

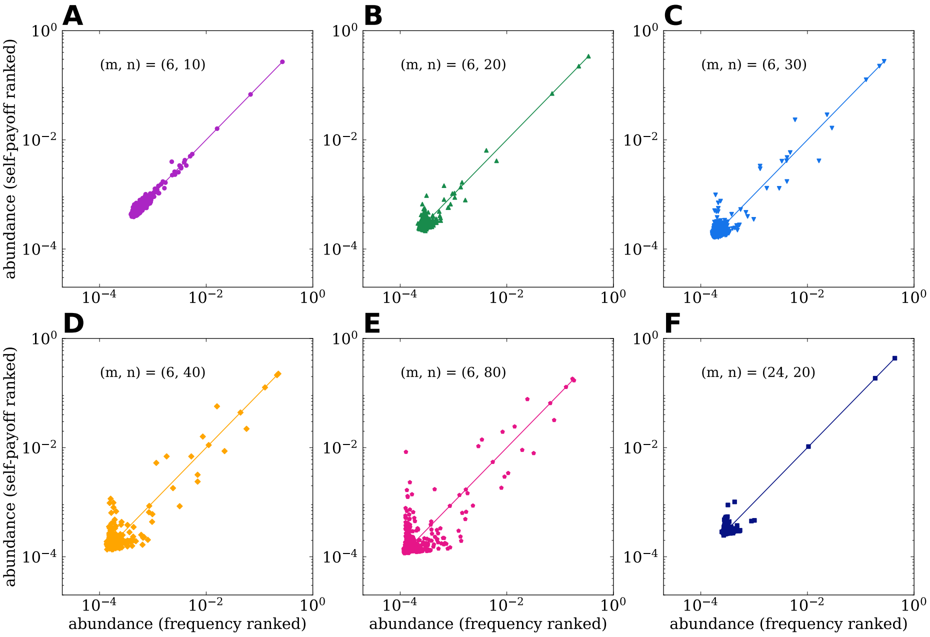

Figure S9 Increasing group size n leads to a lower correlation between type abundance and self-payoff. Each panel shows the average abundance of types ranked against both their average abundance and their self-payoff for populations with constant number of groups $m = 6$ and varying group sizes n. As the group size n increases, the correlation between type abundance and self-payoff decreases. To highlight the importance of population structure and not only on the total population size N, panel **F** displays a population with $(m, n) = (24, 20)$, which has the same total population size $N = 480$ as in panel **E**. Averages were taken over the last 15 measurements of the dynamics.

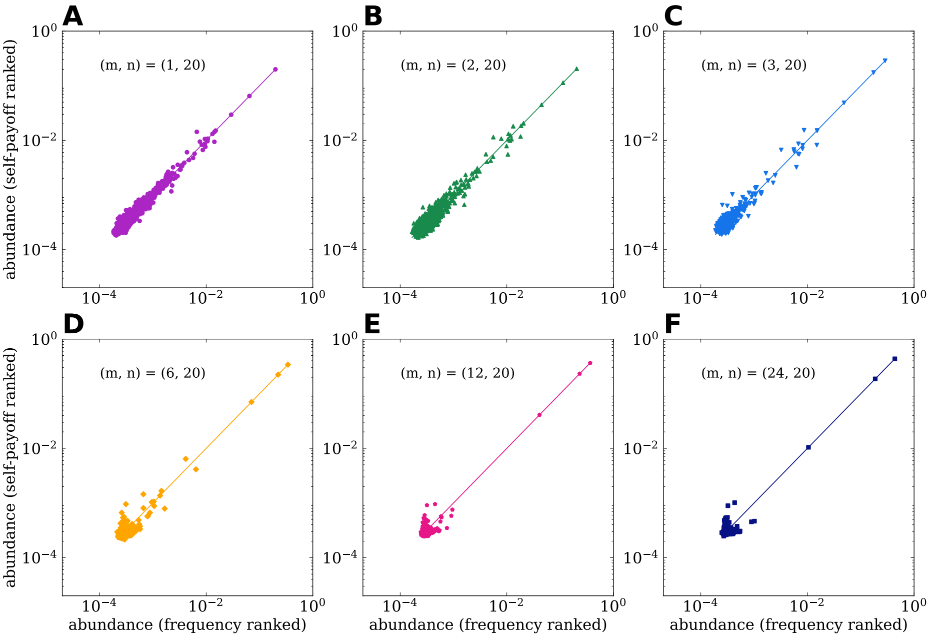

Figure S10 Correlation between type abundance and self-payoff also holds for fixed group sizes $n = 20$ and varying number of groups m. Each panel shows the average abundance of types ranked against both their average abundance and their self-payoff for populations with varying number of groups m and fixed maximum group sizes $n = 20$. In this case, beyond the correlation between both rankings, we note that the abundance of the most abundant type increases with the number of groups m, and that the abundance of all other types becomes more concentrated in lower frequencies. Averages were taken over the last 15 measurements of the dynamics.

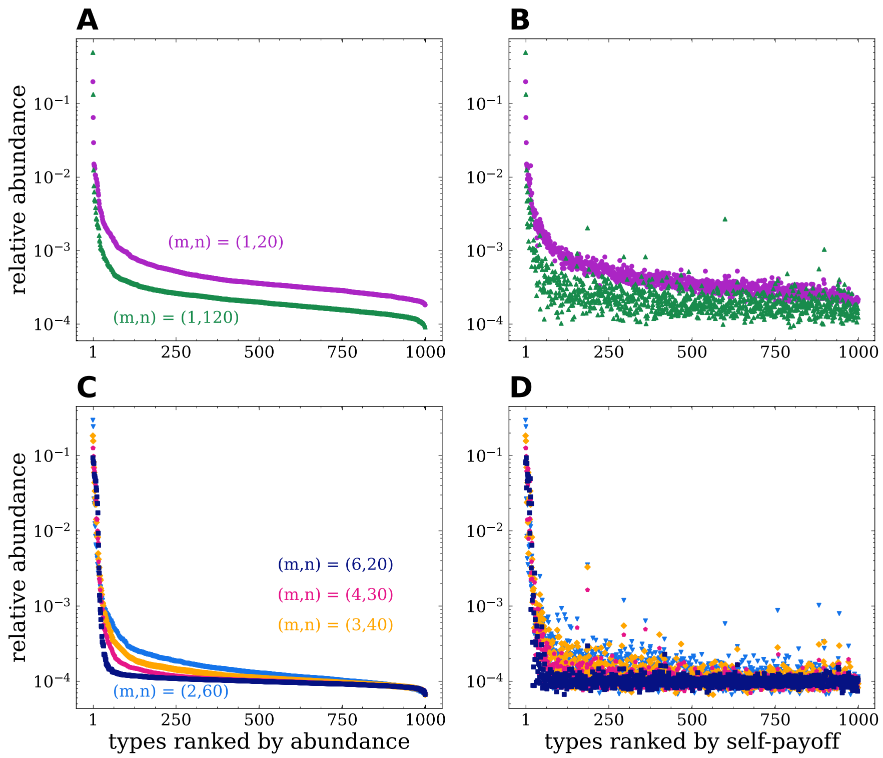

Figure S11 Distribution of ranked average relative abundances for high splitting probability $q = 1.0$ Average relative abundance time-averaged in the long-run (using the last 15 measurements of the dynamics) ranked by the types' relative abundance (left), and by their self-payoff (right). We compare the distributions between single-group populations (top row, same data from main text, Fig. 5) and populations with $N = 120$, $m = 2, 3, 4, 6$ groups (bottom row). When splitting probability is high, types with higher self-payoff tend to be more abundant across different population configurations. This effect is more pronounced as the number of groups m increases, where only a few types are very abundant, while other types are rare. Lower-ranked types typically have lower abundances when compared to the case with $q = 0.001$ or with the single-group distributions. Averages were taken over the last 15 measurements of the dynamics.

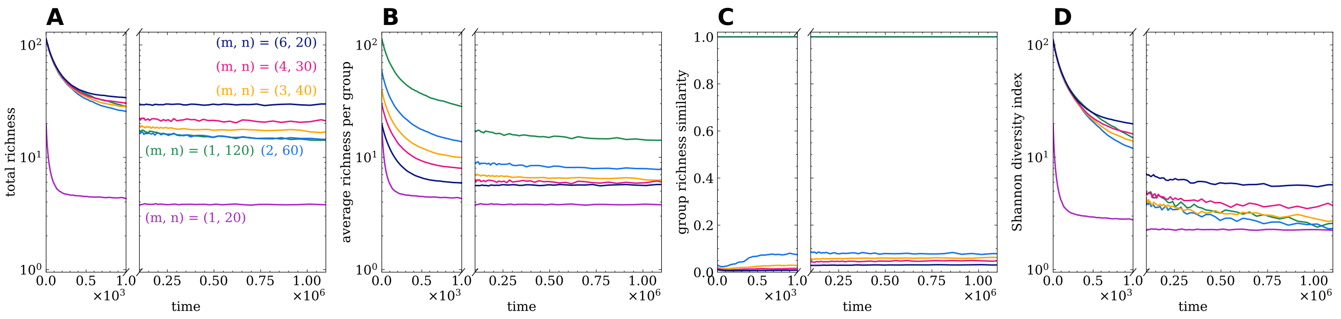

Figure S12 High splitting probability $q = 1.0$ **leads to lower diversity indices.** Different diversity metrics are shown: **A** total richness, **B** average richness per group, **C** group richness similarity, and **D** total Shannon diversity index. Population configurations are: single-group populations with size $(m, n) = (1, 20)$ and $(1, 120)$, and group structured populations with $N = 120$ and splitting probability $q = 1.0$, partitioned as $(m, n) = (2, 60)$, $(3, 40)$, $(4, 30)$, and $(6, 20)$. The description of each diversity index is given in Section Materials and Methods. Population configurations are as described in Fig. S2.

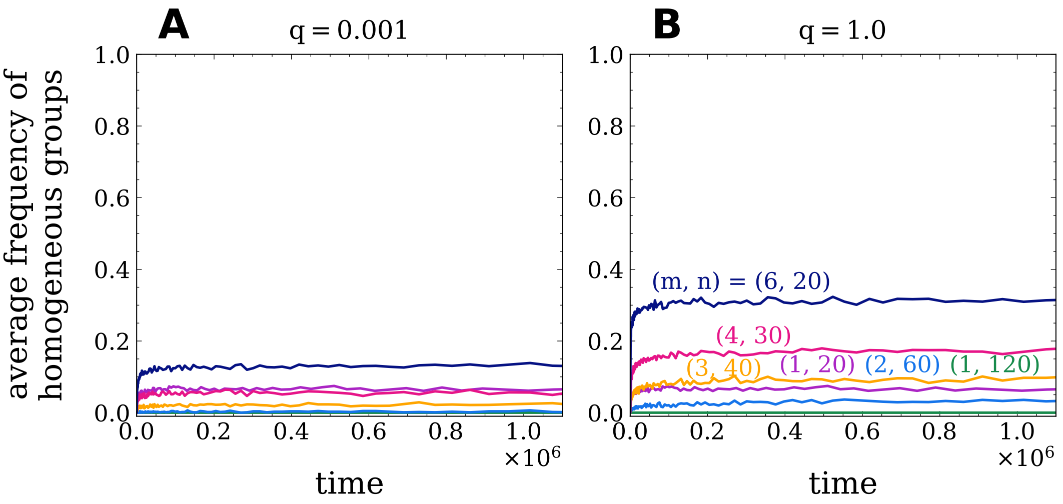

Figure S13 Groups tend to be more homogeneous when splitting probability is high. We compute the average frequency of homogeneous groups for the different (m, n) population configurations: single group ($q = 0.0$) with size $N = 20$ and $N = 120$, and group structured populations with size $N = 120$ and splitting probability **A** $q = 0.001$ and **B** $q = 1.0$ (right), partitioned as $(m, n) = (2, 60)$, $(3, 40)$, $(4, 30)$, and $(6, 20)$. Due to the immigration rate, the large single-group population ($N = 120$) is never homogeneous. The small single-group ($N = 20$) and group structured populations with $q = 0.001$ have a low frequency of homogeneous groups, which increases as the group sizes n decrease and the number of groups m increases. In contrast, the group structured populations with high splitting probability $q = 1.0$ have a higher frequency of homogeneous groups, which is also reflected in the lower group diversity indices (Fig. S12).

References

[1] L. Jost, "Entropy and diversity," *Oikos*, vol. 113, no. 2, pp. 363–375, 2006.

[2] M. Roswell, J. Dushoff, and R. Winfree, "A conceptual guide to measuring species diversity," *Oikos*, vol. 130, no. 3, pp. 321–338, 2021.

[3] L. Jost, "The Relation between Evenness and Diversity," *Diversity*, vol. 2, pp. 207–232, Feb. 2010.

[4] R. H. Whittaker, "Evolution and Measurement of Species Diversity," *TAXON*, vol. 21, no. 2-3, pp. 213–251, 1972.
